# Gallbladder perforation following peroral cholangioscopy‐guided lithotripsy: A case report

**DOI:** 10.1002/deo2.237

**Published:** 2023-04-20

**Authors:** Junichi Kaneko, Moeka Watahiki, Osamu Jindo, Keigo Matsumoto, Toshikatsu Kosugi, Daisuke Kusama, Hiroki Tamakoshi, Tomoyuki Niwa, Yu Takeshita, Masaki Takinami, Ryota Kiuchi, Atsushi Tsuji, Masafumi Nishino, Yurimi Takahashi, Yuzo Sasada, Kazuhito Kawata, Takanori Yamada, Takanori Sakaguchi

**Affiliations:** ^1^ Division of Gastroenterology Iwata City Hospital Shizuoka Japan; ^2^ Division of Gastrointestinal Surgery Iwata City Hospital Shizuoka Japan; ^3^ Division of Hepatology Iwata City Hospital Shizuoka Japan; ^4^ Department of Internal Medicine II Hamamatsu University School of Medicine Shizuoka Japan

**Keywords:** peroral cholangioscopy, lithotripsy, choledocholithiasis, bile ducts, gallbladder perforation

## Abstract

Peroral cholangioscopy‐guided lithotripsy is highly effective in clearing difficult bile duct stones. It can cause adverse events, such as cholangitis and pancreatitis; however, gallbladder perforation is extremely rare. Herein, we describe the case of a 77‐year‐old woman who developed gallbladder perforation following peroral cholangioscopy ‐guided lithotripsy. She was referred to our hospital to treat multiple large bile duct stones. She underwent peroral cholangioscopy‐guided lithotripsy because of conventional lithotripsy failure. After a cholangioscope was advanced into the bile duct, saline irrigation was used for visualization. Electronic hydraulic lithotripsy was performed, but it took time for fragmentation because the calculus was hard. The 2‐h endoscopic procedure did not completely remove the stone, and treatment was discontinued after placing a biliary plastic stent and nasobiliary tube. After the endoscopic procedure, she started experiencing right hypochondrial pain, which worsened the next day. Computed tomography showed a gallbladder wall defect in the gallbladder fundus with pericholecystic fluid. She was diagnosed with gallbladder perforation and underwent emergency surgery. A perforation site was found at the gallbladder fundus. Open cholecystectomy, choledochotomy, and extraction of residual bile duct stones were performed. The patient was discharged 9 days post‐surgery without any complications. The saline irrigation used for visualization may have caused a surge in intra‐gallbladder pressure, resulting in gallbladder perforation. Therefore, endoscopists may need to conserve irrigation water during peroral cholangioscopy‐guided lithotripsy.

## INTRODUCTION

Peroral cholangioscopy (POCS)‐guided lithotripsy is highly effective in clearing difficult bile duct stones.^1^, ^2^, ^3^, ^4^, ^5^ Several adverse events (AEs) of POCS‐guided lithotripsy have been reported, including cholangitis and pancreatitis.^2^, ^3^, ^4^, ^5^ Gallbladder perforation is a rare but life‐threatening complication of acute cholecystitis with a reported mortality rate of 12.5%.^6^ Gallbladder perforation following POCS‐guided lithotripsy has not been reported thus far. Here, we present a case of gallbladder perforation following POCS‐guided lithotripsy.

## CASE REPORT

A 77‐year‐old Japanese woman with hypertension and a history of appendectomy presented to a hospital with nausea and epigastric pain. Abdominal computed tomography (CT) showed multiple common bile duct stones (CBDSs), and she was diagnosed with acute cholangitis. She underwent endoscopic retrograde cholangiopancreatography (ERCP); however, biliary cannulation was unsuccessful. Therefore, the patient was referred to our hospital.

**FIGURE 1:**
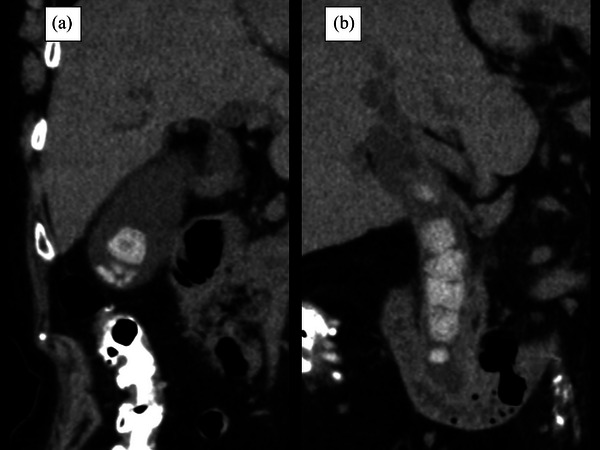
Abdominal computed tomography showing (a) a gallstone measuring 18 mm in diameter and (b) multiple common bile duct stones measuring up to 20 mm in diameter.

Abdominal CT at our hospital showed multiple CBDSs up to 20 mm in diameter and a gallstone up to 18 mm in diameter, with no cholecystitis (Figure 1). At our hospital, ERCP was performed, and biliary cannulation was successful. A cholangiogram showed multiple large CBDSs. Subsequently, endoscopic sphincterotomy and biliary drainage were performed using plastic stents. On day 10 of admission, the patient was discharged without AEs. One month later, the patient was readmitted to our hospital for CBDSs removal.

**FIGURE 2:**
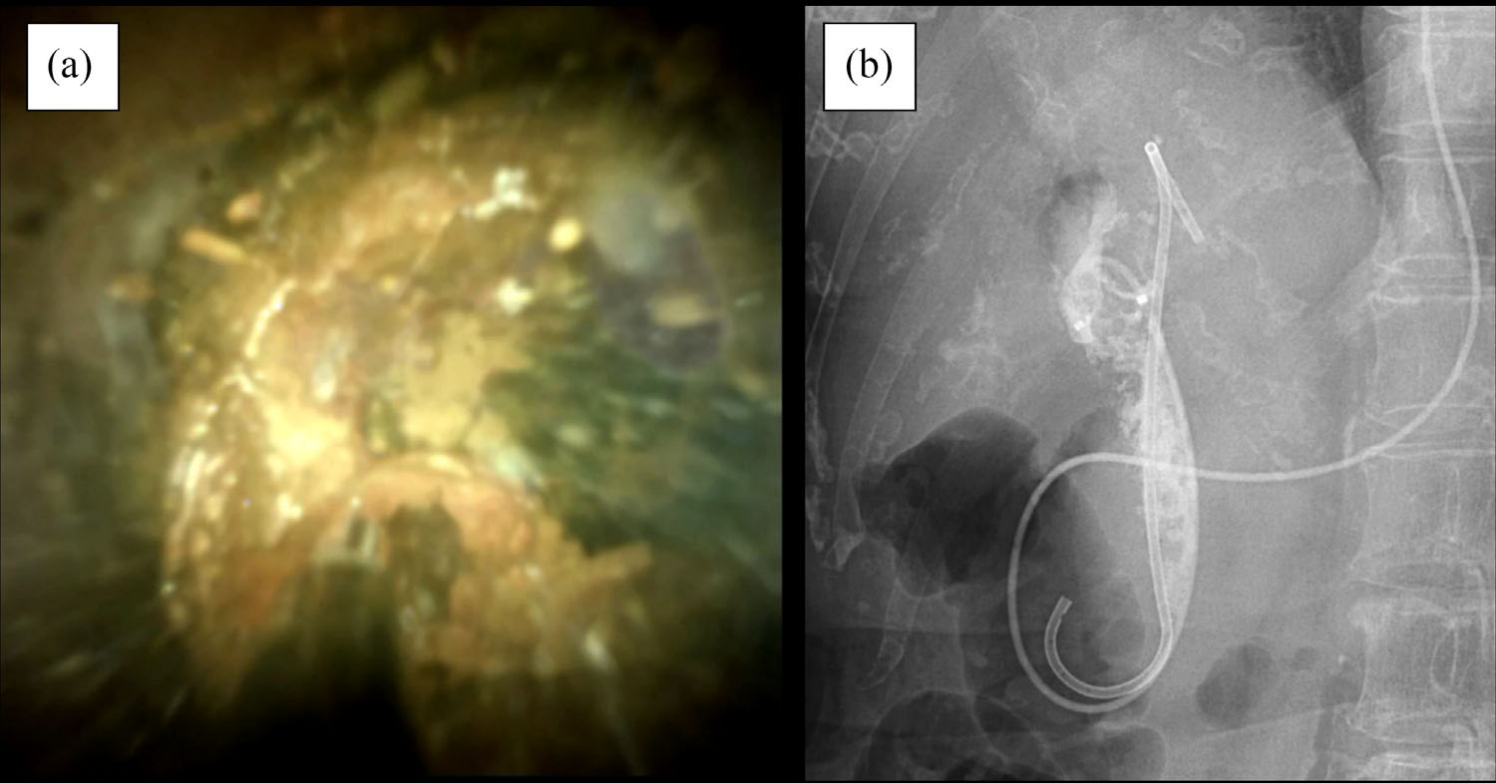
Peroral cholangioscopy‐guided lithotripsy: (a) peroral cholangioscopic findings showing a huge biliary stone; (b) Cholangiography after placement of a plastic stent and nasal biliary tube showing a fragmented fine residual stone in the common bile duct.

Endoscopic papillary large‐balloon dilatation was performed using a 12–14‐mm balloon catheter; mechanical lithotripsy was also performed. However, CBDSs removal was unsuccessful. Therefore, we performed electrohydraulic lithotripsy with POCS guidance. A duodenoscope was advanced into the second part of the duodenum under conscious sedation. A cholangioscope (SpyGlass; Boston Scientific, Marlborough, MA, USA) was advanced over the guidewire through the working channel of the duodenoscope. An irrigation pump and tubing were used for saline irrigation, and the flow rate was controlled using a foot switch. The irrigation volume was set at 100 ml/min (20%). Stone fragmentation with electrohydraulic lithotripsy and stone removal using a basket catheter were alternated. The CBDSs were multiple, hard, and large; therefore, a long‐term endoscopic procedure was needed. After 2 h, fragmentation of the gross stone was successful, but complete stone removal was not achieved. An endoscopic nasal biliary drainage tube and a biliary plastic stent were placed, and this endoscopic procedure was discontinued (Figure 2). A total of 1000 ml of saline was used for irrigation.

**FIGURE 3:**
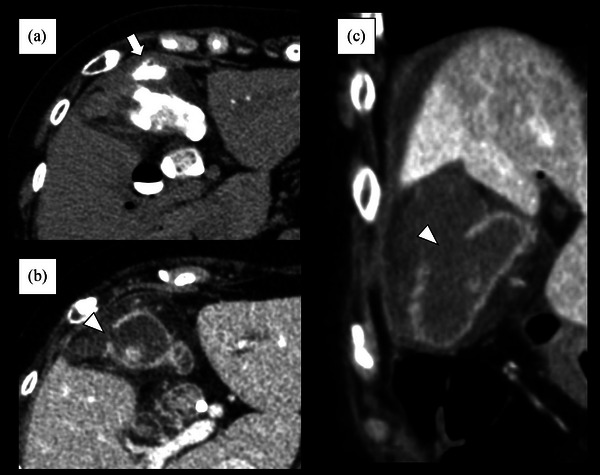
Findings of computed tomography performed after peroral cholangioscopy‐guided lithotripsy; (a) Plain CT images taken immediately after peroral cholangioscopy‐guided lithotripsy showing leakage of contrast agent into the subserous space of the gallbladder wall (white arrow) and marked edematous thickening of the gallbladder wall; (b,c) Contrast computed tomography images taken the day after peroral cholangioscopy‐guided lithotripsy showing a gallbladder wall defect (white arrowhead) in the gallbladder fundus with pericholecystic fluid.

The patient complained of abdominal pain immediately after the endoscopic procedure. Physical examination revealed tenderness in the right hypochondrium, without rebound tenderness or muscular guarding. Immediately after the endoscopic procedure, abdominal plain CT showed leakage of contrast agent into the subserous space of the gallbladder wall and marked edematous thickening of the gallbladder wall (Figure 3a). After the endoscopic procedure, her symptoms temporarily lessened, but worsened the next morning. Physical examination revealed that she developed rebound tenderness and muscular guarding of the right hypochondrium. Peripheral blood analysis revealed a white blood cell count of 7500 cells/μl, total bilirubin level of 1.9 mg/dl, aspartate aminotransferase level of 78 IU/L, and C‐reactive protein level of 8.09 mg/dl. Contrast CT revealed a gallbladder wall defect in the gallbladder fundus with pericholecystic fluid (Figure 3b,c).

**FIGURE 4:**
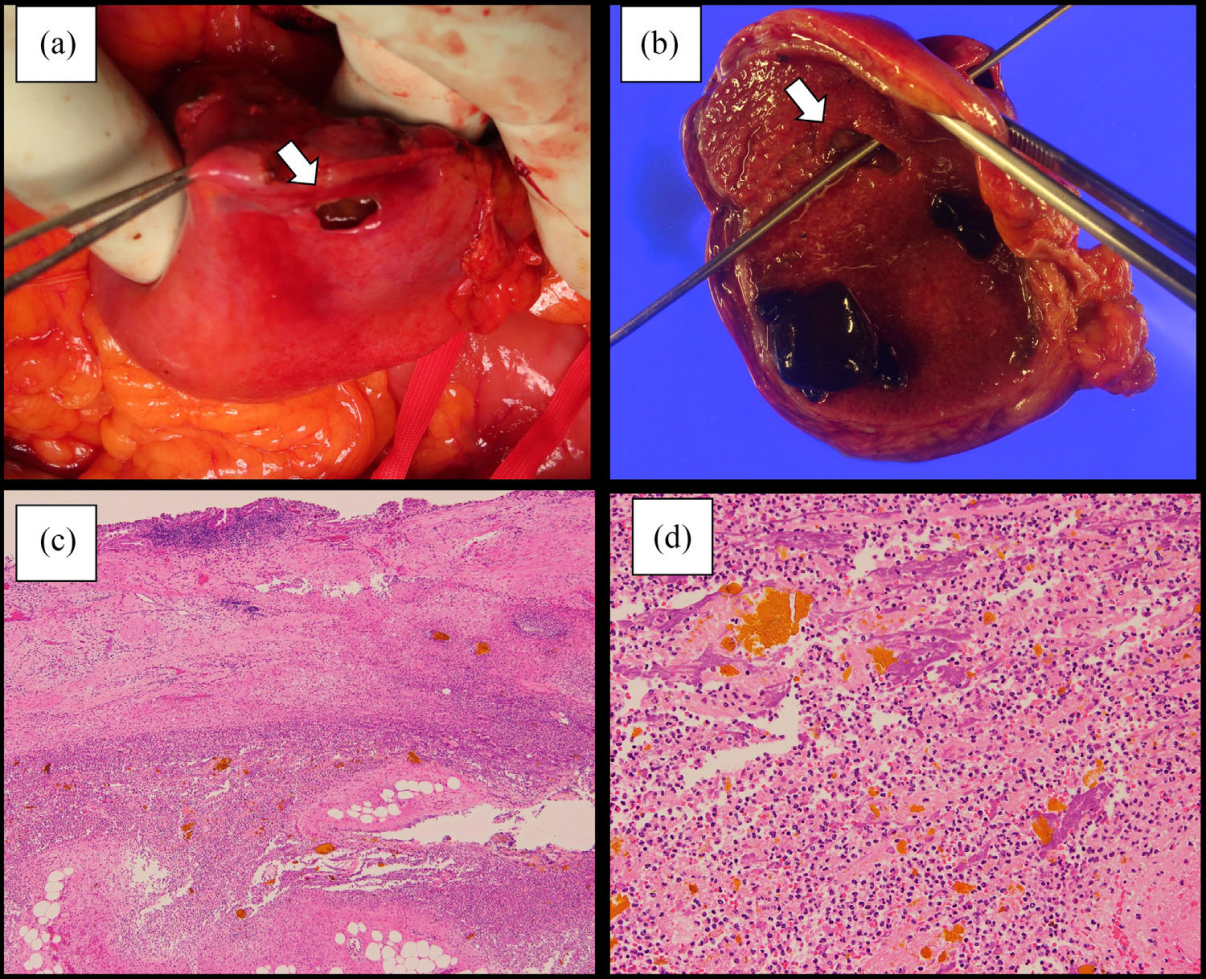
Surgical specimen and pathological findings: (a) the perforation site (1.0 × 1.0 cm) of the gallbladder fundus (white arrow); (b) the surgical specimen showing the perforation site at the gallbladder fundus (white arrow); (c) microscopic findings showing abscess formation around the perforation site (H&E staining, × 4); (d) microscopic findings showing neutrophil infiltration around the perforation site (H&E staining, × 20).

The patient was diagnosed with gallbladder perforation and underwent emergency open surgery. Intraoperatively, a perforation site (1.0 × 1.0 cm) was found at the gallbladder fundus with bile leakage in the space between the gallbladder and liver (Figure 4a). Open cholecystectomy, choledochotomy, and extraction of residual CBDSs were performed. The surgical specimen showed that the perforation site was the fundus of the gallbladder (Figure 4b). Pathological findings revealed neutrophil infiltration and abscess formation around the perforation site (Figure 4c,d) and chronic cholecystitis, such as fibrosis and lymphocytic infiltration, at the other gallbladder wall, with no evidence of malignancy. The patient was discharged 9 days after surgery without complications. Three months later, she underwent ERCP to confirm the absence of residual CBDS and for the removal of the biliary plastic stent.

## DISCUSSION

In the present case, the patient developed gallbladder perforation after undergoing POCS‐guided lithotripsy for multiple large, hard CBDSs. Emergency surgery was performed, and the patient recovered without postoperative complications.

Endoscopic stone extraction is the standard treatment for CBDSs.^1^ Many cases are successfully managed with biliary sphincterotomy and stone extraction using a balloon or a basket catheter. However, difficult bile duct stones, which are large, multiple, and associated with anatomical factors, are challenging to remove using conventional techniques. Therefore, multiple procedures and additional interventional techniques may be required. POCS‐guided lithotripsy has recently become available and is reportedly effective in clearing difficult bile duct stones.^1^, ^2^, ^3^, ^4^ Conversely, it has the potential for AEs with an incidence rate of 2%–22%.^2^, ^3^, ^4^, ^5^ Cholangitis is the most common AE, followed by pancreatitis. However, gallbladder perforation following POCS‐guided lithotripsy has not been reported thus far. To our knowledge, this is the first report of gallbladder perforation following POCS‐guided lithotripsy.

Gallbladder perforation is a rare but potentially fatal disease observed in patients with cholecystitis.^5^ The perforation site was the gallbladder fundus in approximately 60% of the cases. Because the fundus is the most distal part of the gallbladder, its blood supply is compromised. Gallbladder distension can lead to a decreased venous and lymphatic return, leading to ischemia and necrosis of the gallbladder wall, resulting in gallbladder perforation. Gallbladder perforation commonly results from persistent cystic duct obstruction by an impacted stone. Additionally, infections, malignancies, trauma, drugs (e.g., corticosteroids), and systemic diseases such as diabetes mellitus and atherosclerotic heart disease are predisposing factors.^7^

POCS is an extremely rare cause of gallbladder perforation, with only one case reported. Chang et al. reported a case wherein gallbladder perforation developed after POCS‐guided biopsy for biliary strictures; they speculated that irrigation water for cholangioscopy accumulated in the gallbladder instead of the bile duct, causing gallbladder rupture.^8^ In POCS‐guided lithotripsy, irrigation of the bile duct using saline is mandatory for its visualization. Additionally, multiple, large, and hard CBDSs require lengthy procedures for their fragmentation and removal.

In the present case, because the gallbladder wall was not physically irritated by electrohydraulic lithotripsy or mechanical lithotripter, we assumed that the irrigation water was the main cause of gallbladder perforation. Thus, irrigation water used for the long procedure did not flow out of the bile duct due to the CBDSs, which caused inflow to the gallbladder and a surge in intra‐gallbladder pressure, resulting in gallbladder perforation. Therefore, during POCS‐guided lithotripsy, attention should be paid to the amount and flow of irrigation water to avoid prolonged use. However, the patient's background and the amount of irrigation water that predisposes to gallbladder perforation remain unclear; thus, further case accumulation is needed.

In 1934, Niemeier classified free gallbladder perforations into three types: type I, chronic perforations with fistulous communication between the gallbladder and some other viscus; type II, subacute perforations where the perforated gallbladder is surrounded by an abscess walled off by adhesions from the general peritoneal cavity; and type III, acute perforation of the gallbladder into the free peritoneal cavity without protective adhesions.^9^ Emergency surgery is required to treat generalized biliary peritonitis (type III). Cholecystoenteric fistulae (type I) can be managed with urgent or scheduled surgery, depending on the patient's symptomatology. However, the management of localized perforations (type II) remains controversial. Open cholecystectomy for Niemeier type II gallbladder perforation reduces the need for further surgical procedures and postoperative complications but increases the duration of hospital stay.^10^ In the present case, because the patient's symptoms worsened, emergency open surgery was deemed appropriate.

Gallbladder perforation is an extremely rare AE of POCS‐guided lithotripsy, but it can be fatal. Endoscopists may need to conserve irrigation water during POCS‐guided lithotripsy to prevent gallbladder perforation following the procedure.

## CONFLICT OF INTEREST STATEMENT

The authors declare no conflict of interest.

## ETHICS STATEMENT

Not applicable.

## INFORMED CONSENT STATEMENT

Informed consent was obtained from the patient for the publication of this case report and any accompanying images.

## References

[1] Manes G , Paspatis G , Aabakken L *et al.* Endoscopic management of common bile duct stones: European Society of Gastrointestinal Endoscopy (ESGE) guideline. Endoscopy 2019; 51: 472–91. 30943551 10.1055/a-0862-0346

[2] Buxbaum J , Sahakian A , Ko C *et al.* Randomized trial of cholangioscopy‐guided laser lithotripsy versus conventional therapy for large bile duct stones (with videos). Gastrointest Endosc 2018; 87: 1050–60. 28866457 10.1016/j.gie.2017.08.021

[3] Maydeo AP , Rerknimitr R , Lau JY *et al.* Cholangioscopy‐guided lithotripsy for difficult bile duct stone clearance in a single session of ERCP: Results from a large multinational registry demonstrate high success rates. Endoscopy 2019; 51: 922–9. 31250408 10.1055/a-0942-9336

[4] Minami H , Mukai S , Sofuni A *et al.* Clinical outcomes of digital cholangioscopy‐guided procedures for the diagnosis of biliary strictures and treatment of difficult bile duct stones: A single‐center large cohort study. J Clin Med 2021; 10: 1638. 33921514 10.3390/jcm10081638 PMC8069886

[5] Murabayashi T , Ogawa T , Koshita S *et al.* Peroral cholangioscopy‐guided electrohydraulic lithotripsy with a SpyGlass DS versus a conventional digital cholangioscope for difficult bile duct stones. Intern Med 2020; 59: 1925–30. 32389946 10.2169/internalmedicine.4463-20 PMC7492117

[6] Derici H , Kara C , Bozdag AD , Nazli O , Tansug T , Akca E . Diagnosis and treatment of gallbladder perforation. World J Gastroenterol 2006; 12: 7832–6. 17203529 10.3748/wjg.v12.i48.7832 PMC4087551

[7] Gunasekaran G , Naik D , Gupta A *et al.* Gallbladder perforation: A single center experience of 32 cases. Korean J Hepatobiliary Pancreat Surg 2015; 19: 6–10. 26155270 10.14701/kjhbps.2015.19.1.6 PMC4494096

[8] Chang AT , Huang WH . Cholangioscopy complicated by gallbladder perforation. Gastrointest Endosc 2019; 89: 1064–5. 30639541 10.1016/j.gie.2019.01.007

[9] Niemeier OW . Acute free perforation of the gall‐bladder. Ann Surg 1934; 99: 922–4. 17867204 10.1097/00000658-193499060-00005 PMC1390061

[10] Quiroga‐Garza A , Alvarez‐Villalobos NA , Angeles‐Mar HJ *et al.* Localized gallbladder perforation: A systematic review of treatment and prognosis. HPB 2021; 23: 1639–46. 34246546 10.1016/j.hpb.2021.06.003

